# Imaging Activated-T-Lymphocytes in the Salivary Glands of Patients with Sjögren’s Syndrome by ^99m^Tc-Interleukin-2: Diagnostic and Therapeutic Implications

**DOI:** 10.3390/jcm11154368

**Published:** 2022-07-27

**Authors:** Giuseppe Campagna, Luz Kelly Anzola, Michela Varani, Chiara Lauri, Guido Gentiloni Silveri, Lorenzo Chiurchioni, Francesca Romana Spinelli, Roberta Priori, Fabrizio Conti, Alberto Signore

**Affiliations:** 1 Nuclear Medicine Unit, Department of Medical-Surgical Sciences and of Translational Medicine, “Sapienza” University, 00161 Rome, Italy; gius.campagna@gmail.com (G.C.); varanimichela@gmail.com (M.V.); chiara.lauri@uniroma1.it (C.L.); guido.gentiloni@hotmail.it (G.G.S.); lorenzo.chiurchioni@gmail.com (L.C.); 2 Nuclear Medicine, Clinica Colsanitas, Bogotà 110221, Colombia; lkanzola@gmail.com; 3 Rheumatology Unit, Department of Applied Clinical and Medical Therapy, Faculty of Medicine and Pharmacy, “Sapienza” University, 00161 Rome, Italy; francescaromana.spinelli@uniroma1.it (F.R.S.); roberta.priori@uniroma1.it (R.P.); fabrizio.conti@uniroma1.it (F.C.)

**Keywords:** Sjögren’s syndrome, ^99m^Tc-interleukin-2, activated lymphocytes, salivary glands, inflammation imaging

## Abstract

Background: Sjögren’s syndrome (SS) is a progressive autoimmune disease characterized by local mononuclear cell infiltration of the salivary and lachrymal glands. Labial biopsy demonstrates local infiltration by Th1 cells that produce pro-inflammatory cytokines, such as interleukin-2 (IL2). The aim of this study was to assess the utility of ^99m^Tc-labelled-IL2 (^99m^Tc-IL2) in evaluating in vivo the extent and severity of lympho-mononuclear cell infiltration in the salivary glands of patients with SS. Methods: We investigated 48 patients with primary SS and 27 control subjects using ^99m^Tc-IL2 scintigraphy. Furthermore, in a subgroup of 30 patients, we also performed ^99m^Tc-pertechnetate scintigraphy (^99m^TcO_4_^−^) for evaluation of the salivary gland function. Results: ^99m^Tc-IL2 uptake in the salivary glands of SS patients was higher than in the control subjects (1.30 ± 0.16 vs. 0.83 ± 0.08 for parotids and 1.36 ± 0.15 vs. 1.16 ± 0.07 for submandibular glands; *p* < 0.0001). The salivary gland uptake of ^99m^Tc-IL2 in patients with a longer history of disease was lower compared with the recently diagnosed patients. A significant direct correlation was found between the uptake of ^99m^Tc-IL2 and histology. Conclusions: ^99m^Tc-IL2 scintigraphy showed that the degree of lymphocytic infiltration of major salivary glands is variable in patients with different disease durations. Patients with a high ^99m^Tc-IL2 uptake could be efficiently treated with immuno-modulatory drugs and the efficacy of treatment could be followed-up by ^99m^Tc-IL2 scintigraphy.

## 1. Introduction

Sjögren’s syndrome (SS) is a chronic inflammatory disease of the lachrymal and salivary glands causing keratoconjunctivitis sicca (KCS) and xerostomia [1]. SS is the second immunological disease in frequency and women are more affected compared with men (9:1) [2]. Nowadays, the diagnosis of SS is based on clinical, serological, and instrumental parameters, such as the presence of KCS, xerostomia, auto-antibodies, and positive sub-labial salivary gland (SSG) biopsy [3,4]. Of these, the most important criteria are the histological findings, including acinar atrophy, fibrosis, ductal changes, and focal lymphocytic infiltrations in the minor salivary glands’ tissues [5].

Lymphocytic infiltration is initially characterised by activated T-lymphocytes of the CD4+ phenotype (Th1), which produce a large amount of mRNA for interleukn-2 (IL2), interferon-alpha (IFN-a), and interleukin-10 (IL10) [6]. These cytokines cause tissue damage, ultimately evolving in the fibrosis of salivary glands, and causing complete loss of functionality. This process is signal-mediated through the T-cell receptor that interacts with class II antigens on the epithelial cells of exocrine glands. In particular, T-cells become activated and express a high quantity of IL2 receptors (I2R), which are surface hetero-trimeric proteins that bind IL2. The IL2−IL2R interaction causes the activation, differentiation, and growth of various immunological cells [7,8]. In particular, IL2R has been found on the surface of activated T-cells, but also in some B-cells and macrophages. Therefore, IL2R is considered an early marker of T-lymphocyte activation [9]. In the following phases of disease progression, Th2 and Th17 cells may appear with the production of other cytokines, as well as the so-called follicular helper T-cells that seem to play a protective role [10].

IL2 labelled with ^123^I (^123^I-IL2) and ^99m^Tc (^99m^Tc-IL2) has been used in vivo to detect activated lymphocytes in different autoimmune diseases, such as Coeliac disease [11], IDDM [12], Hashimoto’s thyroiditis, Takayasu’s arteritis [13], multiple-autoimmunity [14] and, recently, to detect immune infiltration on cancer [15]. We also demonstrated that ^99m^Tc-IL2 allows for planning specific immunotherapy in patients with active disease and the non-invasive follow-up of patients [16]. Interestingly, we observed that patients with thyroid autoimmune diseases (i.e., Graves’ disease or Hashimoto thyroiditis) may show ^99m^Tc-IL2 uptake in the salivary glands, predicting the development of a secondary Sjögren’s syndrome in the following years [17,18].

The aim of our study was to evaluate the uptake of ^99m^Tc-IL2 in the salivary glands as an indicator of inflammation. We also studied its relationship with other clinical, serological, histological, and functional parameters, such as salivary gland scintigraphy with ^99m^Tc-sodium pertechnetate (^99m^TcO_4_^−^) [19].

## 2. Materials and Methods

### 2.1. Study Design

This is a prospective, open study. The study protocol was approved by the ethics committee of the University of Rome, “Sapienza”. All patients and normal volunteers gave written informed consent.

### 2.2. Patients

We studied 48 patients with SS (36 women and 12 men; mean age 50.78 ± 13.08 years (95% CI 46.59 to 54.96) in whom the disease was diagnosed based on the fulfilment of at least four of the six European Community Study Group’s criteria, and by labial salivary gland biopsys [4]. In all patients, we excluded other processes that may have caused sicca syndrome as well as the absence of other systemic autoimmune diseases. We compared our patients with 27 sex and age-matched control oncological subjects (14 women and 13 men; age range 35–83 years), with no evidence of autoimmune diseases, who performed the ^99m^Tc-IL2 scintigraphy for investigating the presence of T-cell infiltrates in tumor lesions.

### 2.3. Clinical and Laboratory Assessments

All patients complained about a subjective feeling of ocular or oral dryness (Table 1). Nevertheless, xerophthalmia was objectively assessed by the Schirmer and/or Break Up Time (BUT) tests. Oral involvement was studied by salivary gland scintigraphy with ^99m^Tc-^99m^TcO_4_^−^ in 30 patients. Histopathological examination of the labial salivary glands, accepted as the gold standard in primary Sjogren’s syndrome [20], was performed in 28 patients. The standard grading criteria [21] ranged from 0 to 4 based on the presence of mononuclear cell infiltration with a periductal distribution. This is a typical finding in patients with primary SS, compared with the perivascular infiltration that occurs mainly in secondary SS.

We also tested the presence of antinuclear antibodies (ANA) and anti-extractable nuclear antigen-antibodies (ENA) by indirect immunofluorescence, and auto-antibodies anti-Ro (SSA) and anti-La (SSB) by immune-electrophoresis [22].

### 2.4. Salivary Gland Scintigraphy

Both ^99m^Tc-IL2 and ^99m^TcO_4_^−^ scintigraphy were performed in patients at a time interval of 1 to 5 weeks.

#### 2.4.1. ^99m^Tc-IL2 Scintigraphy

Human recombinant IL2 (Proleukin^®^, Novartis, Basel, Switzerland) was labelled using the method described by Chianelli et al. [23]. Scintigraphy was performed in all patients and control subjects after i.v. injection of 111–185 MBq of ^99m^Tc-IL2. Planar anterior images (256 × 256 pixel matrix) of the neck were acquired 45 min after injection. Quantitative analysis of the planar images was carried out by drawing an irregular region of interest (ROI) over the parotid and submandibular glands. The background was calculated with a rectangular ROI drawn below the two submandibular glands and above the thyroid region.

**Figure 1:**
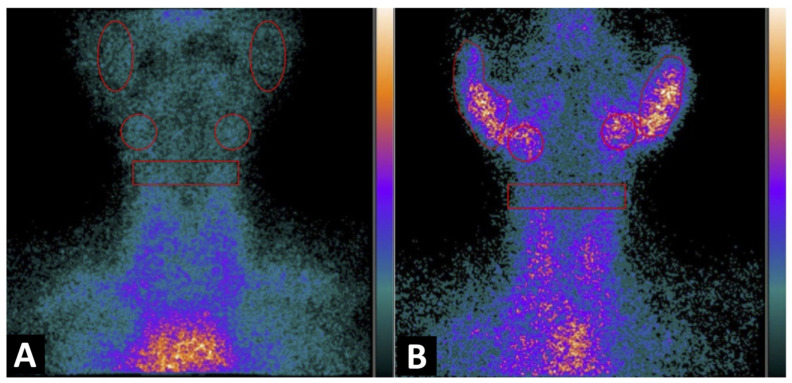
Planar image of the neck obtained 1h after ^99m^Tc-IL2 injection in a control subject (**A**) and in a patient with Sjögren syndrome at time of diagnosis (**B**). In (**A**) the scan shows no ^99m^Tc-IL2 uptake by the salivary glands. In (**B**) an evident accumulation of ^99m^Tc-IL2 can be observed in both parotids and submandibular glands, indicating the presence of activated lymphocytes. The calculated parotid to background (P/B) ratios are 1.35 and 1.30 in right and left glands, respectively, and the submandibular gland to background (S/B) ratios are 1.57 and 1.64 in right and left glands, respectively.

In the control subjects and when the salivary glands were not detectable, a circular ROI was drawn corresponding to the parotid or submandibular region (Figure 1).

The results of the quantitative analysis were expressed as the parotid to background (P/B) ratio and submandibular to background (S/B) ratio, after calculating the mean uptake between the two contralateral glands.

#### 2.4.2. ^99m^TcO_4_^−^ Scintigraphy

Dynamic salivary gland scintigraphy was performed in 30 patients, after i.v. injection of 80–150 MBq ^99m^TcO_4_^−^, with a gamma camera equipped with a parallel-hole, low energy, high sensitivity collimator, and 140 keV photopeak for the technetium. Anterior sequential images were acquired at 1 s per frame for 1 min (vascular phase) and 10 s per frame for the next 24 min. Fifteen minutes after the injection, 3 mL of lemon juice was administered orally as a stimulus, as described by Bohulaslavizki et al. [24] and Anjos et al. [25].

Data analysis of the acquired images was performed by drawing irregular ROIs on each parotid and oval-shaped regions over each submandibular gland.

For the background regions, we drew two ROIs on the temporal regions [24].

The quantitative analysis included the evaluation of the ^99m^Tc-pertechnetate ejection fraction (EF), which was calculated with the following equation:

EF(%) = [(U12-14-U18-20) × 100]/U12-14.(1)

**Figure 2:**
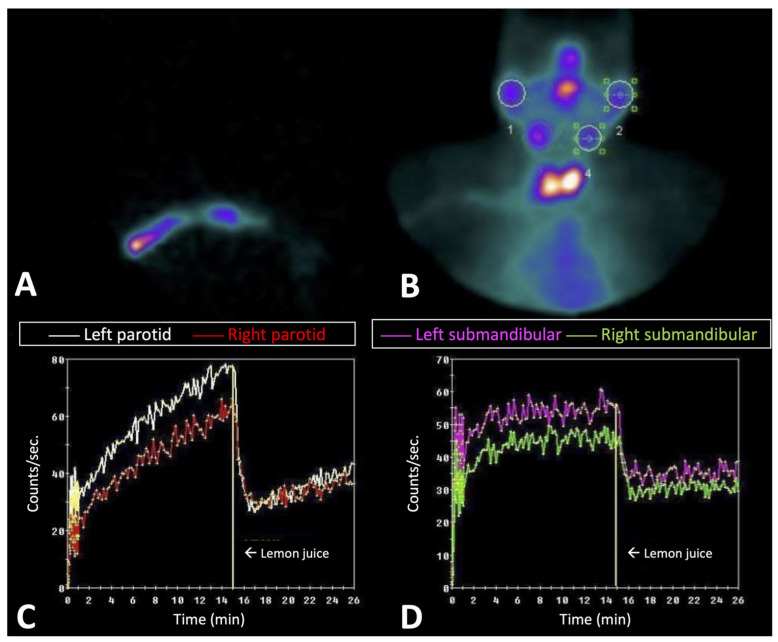
Dynamic study with ^99m^TcO_4_^−^ in patients with Sjögren syndrome 3 years after diagnosis. (**A**,**B**) show the start of dynamic images and the summary of all dynamic images, respectively. (**C**,**D**) show the quantitative analysis of the parotid and submandibular glands, respectively. A moderate accumulation of ^99m^TcO_4_^−^ can be observed in the parotids and, to a lesser extent, in the submandibular glands, indicating the presence of a residual function.

The maximum ^99m^TcO_4_^−^ uptake was calculated as the percentage of the injected dose (ID), as described by Anjos et al. (Figure 2) [25]. A positive scintigraphy was considered when the EF or maximum uptake was lower than the lower value of the confidence interval of the normal subjects.

### 2.5. Statistical Analysis

Continuous variables were shown as mean ± standard deviation with a 95% CI (confidence interval). Categorical variables were expressed as absolute frequencies and percentages, as *n* (%). Comparisons between the control subjects vs. Sjögren’s patients of the ^99m^Tc-IL2 uptake in the parotid glands (mean P/B) and ^99m^Tc-IL2 uptake in the submandibular glands (mean P/B) were evaluated using the Student’s test. The normality of these variables was tested using the Shapiro−Wilk test and checking the Q-Q plot. In the presence of heteroscedasticity, we used the correction of Satterthwaite.

The correlation between disease duration and biopsy vs. ^99m^Tc-IL2 uptake in parotid glands (mean P/B), and ^99m^Tc-IL2 uptake in submandibular glands (mean P/B) was evaluated using Kendall’s tau-b (τb), because the normality of the continuous variables analysed not was verified and the confidence intervals were determined using the bootstrapping method. Statistical analysis was performed using SAS v.9.4 and JMP PRO v.16 (Institute Inc., Cary, NC, USA). A *p*-value < 0.05 was considered statistically detectable.

## 3. Results

**Figure 3:**
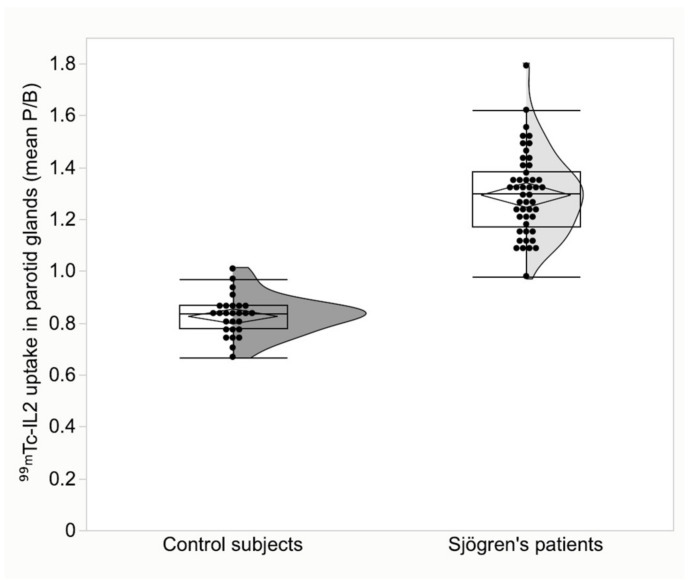
Parotid gland uptake of ^99m^Tc-IL2 in patients with Sjögren disease compared to control subjects (*p* < 0.001).

**Figure 4:**
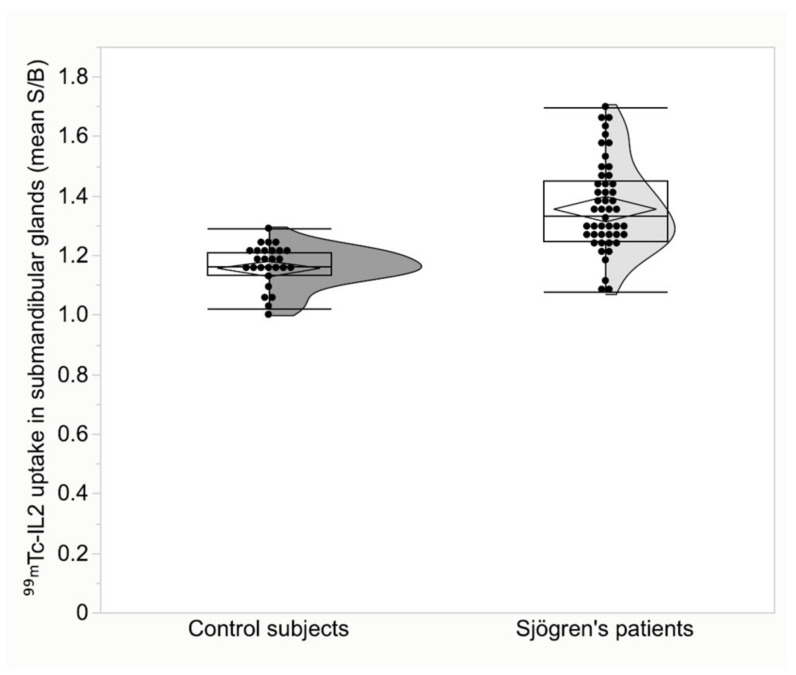
Submandibular gland uptake of ^99m^Tc-IL2 in patients with Sjögren disease compared to control subjects (*p* < 0.001).

The characteristics of the patients and the criteria for the diagnosis of SS are summarized in Table 1. Patients with SS differed significantly from the control subjects. The mean ^99m^Tc-IL2 uptake in parotid glands was 1.30 ± 0.16 (95% CI 1.25 to 1.34) in patients vs. 0.83 ± 0.08 (95% CI 0.80 to 0.86) in the controls, *p* < 0.001 (Figure 3). In the submandibular glands, ^99m^Tc-IL2 uptake was 1.36 ± 0.15 (95% CI 1.31 to 1.40) in patients vs. 1.16 ± 0.07 (95% CI 1.13 to 1.18) in the controls, *p* < 0.001 (Figure 4).

**Figure 5:**
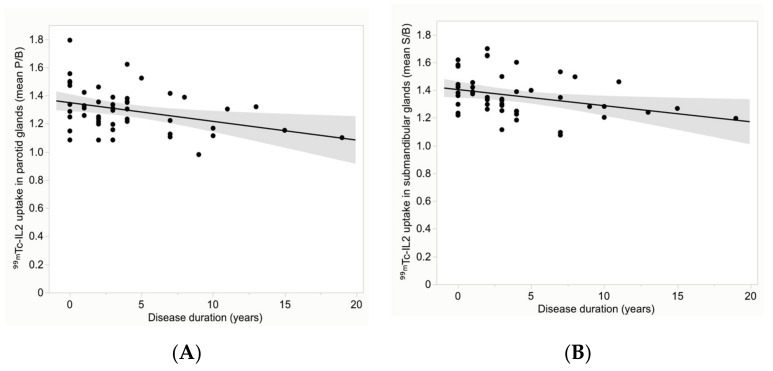
Distribution of the mean ^99m^Tc-IL2 uptake (P/B) in the parotid glands (**A**) and (S/B) in the submandibular glands (**B**) over the disease duration. A significant inverse correlation can be observed in all glands (correlation coefficient is −0.22 (*p* = 0.03) for the parotids and −0.29 (*p* = 0.006) for the submandibular glands).

Interestingly, despite the variability of ^99m^Tc-IL2 uptake in the glands of patients, we found a significant inverse correlation between the mean target/background ratios (P/B and S/B) with disease duration (Figure 5). The correlation coefficient was −0.22 (95% CI −0.43 to −0.02), *p* = 0.03, for the parotids and −0.29 (95% CI −0.47 to −0.09), *p* = 0.006, for the submandibular glands.

### 3.1. Histopathological Tissue Analysis

The histology examination of the minor salivary glands biopsies showed a typical characteristic of SS according to Chisholm e Mason classification in 22 out of 28 patients. Moreover, positive grading (between 3 and 4) was detected in 11 (39.3%) patients while 17 patients (60.7%) resulted with negative biopsy (between 0 and 2) (Table 1).

**Figure 6:**
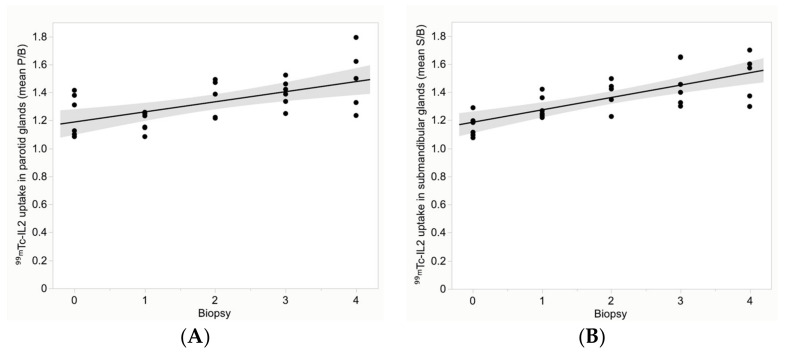
Distribution of the mean ^99m^Tc-IL2 uptake (P/B) in the parotid glands (**A**) and (S/B) in the submandibular glands (**B**) over the biopsy score. A significant correlation can be observed in all glands (correlation coefficient is 0.46 (*p* = 0.001) for the parotids and 0.60 (*p* < 0.0001) for the submandibular glands).

Despite this high variability in biopsies, a significant correlation was found between ^99m^Tc-IL2 uptake and biopsy score, in both parotid and submandibular glands (Figure 6). The correlation coefficient was 0.46 (95% CI 0.18 to 0.68), *p* = 0.001, for the parotids and 0.60 (95% CI 0.35 to 0.76), *p* < 0.0001, for the submandibular glands.

No correlation was found between the biopsy score and quantitative parameters obtained by ^99m^TcO_4_^−^ scintigraphy or with antibody titres.

### 3.2. ^99m^Tc-IL2 Scintigraphy

Through the qualitative analysis, we observed an uptake of ^99m^Tc-IL2 in at least one gland in all of the patients. The semi-quantitative analysis showed that only one patient had only one gland (parotid) with a T/B higher than the maximum T/B found in the controls. Seventeen patients had at least two glands, 10 patients had three glands, and 20 patients had all four glands with a T/B higher than the maximum T/B found in the controls.

### 3.3. ^99m^TcO_4_^−^ Scintigraphy

^99m^TcO_4_^−^ scintigraphy provided two different parameters: the ejection fraction and the maximum uptake of ^99m^TcO_4_^−^ as a percentage of the injected dose. All of the patients had a reduced EF in at least three glands. In contrast, the maximum uptake was reduced in 24/30 patients (80%) when considering at least one functioning gland. We found no correlation between SGS parameters and all of the other variables. In particular, a mild, non-statistically detectable, inverse correlation was found between the SGS parameter and ^99m^Tc-IL2 uptake in the glands. In particular, the EF and ^99m^Tc-IL2 uptake in the parotids showed a correlation coefficient of −0.16 (95% CI −0.42 to 0.17), *p* = 0.21. The maximum uptake and ^99m^Tc-IL2 uptake in the parotids showed a correlation coefficient of −0.10 (95% CI −0.36 to 0.17), *p* = 0.45. EF and ^99m^Tc-IL2 uptake in the submandibular glands showed a correlation coefficient of −0.15 (95% CI −0.40 to 0.14), *p* = 0.24. The maximum uptake and ^99m^Tc-IL2 uptake in the submandibular glands showed a correlation coefficient of −0.12 (95% CI −0.34 to 0.11), *p* = 0.36.

## 4. Discussion

Nowadays, there is no single “gold standard” of oral involvement that is sensitive and specific enough to be the basis for a diagnosis of SS. Moreover, many different diagnostic methods such as sialography, salivary glands scintigraphy with technetium, and lip biopsy have been included into the criteria system of the European Community [3].

Parotid sialography is the most specific (92%–100%) and is accepted as a generally safe method of assessing anatomic changes in SS. However, its usefulness in diagnosis remains questionable for a wide range of sensitivities due to negative results, especially in the early stage of disease [26,27]. Moreover, some technical difficulties and complications related to the procedure have been reported [28]. Some authors have proposed computed tomography (CT) and magnetic resonance imaging (MRI) as alternative accurate methods to detect the parenchymal inhomogeneity characteristic of glands in Sjogren’s syndrome, but both of them are expensive and are not able to show the activity of the disease [29,30]. Other studies have been published on ultrasonography (US) as a non-invasive and safe imaging procedure to obtain information about the morphological changes of salivary glands in primary SS [31,32,33]. More recently, Milic et al. described that patients with primary SS present more frequent pathological changes of the posterior borders, parenchymal inhomogeneity with hypoechogenic areas, and/or hyperechogenic reflections in major salivary glands [34]. Although US is able to follow-up on parenchymal damage, it cannot directly show the grading of inflammation useful in planning treatment.

In the past, salivary glands scintigraphy with ^99m^Tc-pertechnetate have been proposed to evaluate intact salivary gland parenchyma. Its major advantage, compared with other imaging methods, is its ability to present information on parenchymal damage as well as the excretion function of all glands simultaneously. At present, there is no consensus about which quantitative indices are trustworthy for the diagnosis of SS. However, some reports have described that decreased secretion parameters in parotid glands and decreased accumulation of ^99m^Tc in the submandibular glands are highly sensitive indicators of SS [35]. Nevertheless, our data do not support the hypothesis that a decreasing salivary function is correlated with the severity of lymphocytic infiltration. Instead, it seems that there is an inverse correlation between these parameters.

Indeed, although quantitative SGS is sensitive enough to detect abnormalities of parenchyma, it reflects only the gland’s function in SS. Moreover, these alterations can be found in other diseases, and its rate will increase with age, even in healthy subjects. Therefore, SGS provides an overall sensitivity from 62% to 89% because of the early stage of disease and the low grade of damage in salivary glands [36].

The criteria of the European Community consider the lip biopsy to be the most reliable diagnostic test, and it is still most accurate method for the definitive diagnosis of SS. Nevertheless, in some cases, it is not conclusive. The sensitivity and specificity of the lip biopsy range from 82% to 95% and from 75% to 90%, respectively [37]. It also shows false-negative findings in 18–40% of patients and false-positive in 6–9% of healthy subjects [38]. Moreover, patients with myasthenia gravis, sialolithiasis, and other autoimmune diseases with no syndrome sicca may present lymphocytic infiltration in the salivary glands [26]. Furthermore, the lip biopsy cannot be used as a routine procedure in the follow-up of patients, because it is invasive and is not well accepted. Finally, in some patients, in the early stage of disease, the lip biopsy may not show a lymphocytic infiltration [39], which can become detectable in a second sample repeated after 1 year [40]. In addition, the degree of lymphocytic infiltrate may change over time [41]. Moreover, in major and minor salivary glands, there may be different damage and the degree of epithelial cell damage in a salivary gland biopsy does not always correspond to the salivary flow secretion [42]. Overall, the lip biopsy provides limited information that cannot be applied to all major salivary glands and should not be used to select the most appropriate therapy or to follow-up its efficacy over time.

A non-invasive method to detect lymphocytic infiltration in all major glands is thus desirable [43,44]. Our results suggest that ^99m^Tc-IL2 scintigraphy could be used to detect and quantify the lymphocytic infiltration in all major salivary glands. Despite finding a positive correlation between the lip biopsy score and ^99m^Tc-IL2 uptake in major glands, this analysis could be affected by the presence of scores 0–1 and 2 that, indeed, are not related to lymphocytic infiltration. Therefore, larger studies are needed to clearly establish the clinical role of ^99m^Tc-IL2 and the relation of its uptake in glands with different phases of the diseases.

Most of our patients had a recent diagnosis (within 4 years) and only a few patients had a diagnosis between 5 and 20 years. Furthermore, we did not study the patients over time. This can be a limit of our population, thus not allowing us to conclude on the course of lymphocytic infiltration over time, despite finding an inverse correlation between disease duration and ^99m^Tc-IL2 uptake in all of the glands. Another limitation of this study is that biopsy was scored in classes from 0 to 4, and this may not reflect the extent of lymphocytic infiltration in salivary glands. Furthermore, as the process is not synchronous in all glands, a biopsy of submandibular and parotid glands would have certainly been more correct, but ethically impossible to perform. Indeed, if we consider only patients within 2−3 years of diagnosis, there would be a high variability in ^99m^Tc-IL2 uptake in the glands, suggesting that there is a great variability between patients and that the disease is not synchronous in all glands. This finding may have important therapeutic implications.

## 5. Conclusions

In conclusion, our data suggest ^99m^Tc-IL2 scintigraphy can be a new tool to assess lymphocytic infiltration in SS. The gland uptake of ^99m^Tc-IL2 is higher in patients with early diagnosis, from 0 to 1 years of disease, whereas in older diagnoses, the infiltration decreases followed by a fibro-sclerotic process. This scintigraphy could be used to monitor in vivo, non-invasively, the efficacy of immune-modulatory therapies.

## Figures and Tables

**Table 1:** Patient’s characteristics and the criteria for clinical diagnosis.

Patient	Sex	Biopsy	Antibodies	Xerophthalmia	Xerostomia	Schirmer’s Test or BUT	EF or %Max Uptake
1	F	3	pos	yes	yes	pos	pos
2	F		pos	yes	yes		pos
3	F	4	pos	yes	yes		pos
4	F	0	pos	yes	yes		pos
5	F		neg	yes	yes	pos	pos
6	F	0	pos	yes	yes		pos
7	F	0	pos	yes	yes		pos
8	F	4	pos	yes	yes		pos
9	F		pos	yes	yes		pos
10	F	1	neg	yes	yes		pos
11	F	0	pos	yes	yes	pos	pos
12	F		pos	yes	yes		pos
13	F		pos	yes	yes		pos
14	F		pos	yes	yes		pos
15	F	3	pos	yes	no		pos
16	F	2	pos	yes	yes		pos
17	F		pos	yes	yes		pos
18	F		pos	yes	yes		pos
19	F	1	pos	no	yes		pos
20	F	3	pos	no	yes		pos
21	F		pos	yes	yes		pos
22	F	1	pos	yes	yes		pos
23	F	2	neg	yes	yes		pos
24	F		pos	yes	yes		pos
25	M	4	neg	yes	yes		pos
26	F		pos	yes	yes		pos
27	F	4	pos	yes	yes		pos
28	F		pos	yes	yes		pos
29	F	1	pos	yes	yes		pos
30	M	4	neg	yes	yes		pos
31	M		pos	yes	yes		pos
32	F		pos	yes	yes		pos
33	F		pos	yes	yes		pos
34	M	0	neg	yes	yes	pos	pos
35	F		pos	yes	yes		pos
36	F	1	pos	yes	yes		
37	F	0	pos	yes	yes	pos	
38	F	2	pos	yes	yes		
39	F	2	pos	yes	yes		pos
40	F	3	neg	yes	yes		pos
41	F	2	pos	yes	yes		pos
42	F	3	pos	yes	yes		pos
43	F	1	pos	yes	yes		pos
44	F		pos	yes	yes		pos
45	F		pos	yes	yes	pos	
46	F		pos	yes	yes		pos
47	F		pos	yes	yes		pos
48	M	3	pos	yes	yes		pos

Biopsy score according to Chisholm e Mason classification; antibodies = positivity to either SSA or SSB or both antibodies; EF or %max uptake = reduced ejection fraction or reduced maximum ^99m^TcO_4_^−^ uptake of salivary glands at functional scintigraphy. Empty boxes = not performed.

## Data Availability

All data are available upon request to G.C.
